# Microscale grooves regulate maturation development of hPSC‐CMs by the transient receptor potential channels (TRP channels)

**DOI:** 10.1111/jcmm.16429

**Published:** 2021-03-10

**Authors:** Taoyan Liu, Siyao Zhang, Chenwu Huang, Shuhong Ma, Rui Bai, Yanan Li, Yun Chang, Chenwen Hang, Amina Saleem, Tao Dong, Tianwei Guo, Youxu Jiang, Wenjing Lu, Lina Zhang, Luo Jianwen, Hongfeng Jiang, Feng Lan

**Affiliations:** ^1^ Beijing Lab for Cardiovascular Precision Medicine Anzhen Hospital Capital Medical University Beijing China; ^2^ State Key Laboratory of Stem Cell and Reproductive Biology Institute of Zoology Chinese Academy of Sciences Beijing China; ^3^ University of Chinese Academy of Sciences Beijing China; ^4^ Department of Biomedical Engineering School of Medicine Tsinghua University Beijing China; ^5^ Department of Cardiology Peking University Third Hospital Beijing China; ^6^ State Key Laboratory of Chemical Resource Engineering Beijing Laboratory of Biomedical Materials Beijing Advanced Innovation Center for Soft Matter Science and Engineering Beijing University of Chemical Technology Beijing China; ^7^ Key Laboratory of Remodeling‐Related Cardiovascular Diseases Ministry of Education Beijing Anzhen Hospital Capital Medical University Beijing China

**Keywords:** hPSC‐CMs, maturation, microtopography, topographic cues, TRP channels

## Abstract

The use of human pluripotent stem cell‐derived cardiomyocytes (hPSC‐CMs) is limited in drug discovery and cardiac disease mechanism studies due to cell immaturity. Micro‐scaled grooves can promote the maturation of cardiomyocytes by aligning them in order, but the mechanism of cardiomyocytes alignment has not been studied. From the level of calcium activity, gene expression and cell morphology, we verified that the W20H5 grooves can effectively promote the maturation of cardiomyocytes. The transient receptor potential channels (TRP channels) also play an important role in the maturation and development of cardiomyocytes. These findings support the engineered hPSC‐CMs as a powerful model to study cardiac disease mechanism and partly mimic the myocardial morphological development. The important role of the TRP channels in the maturation and development of myocardium is first revealed.

## 1 INTRODUCTION

Human pluripotent stem cell‐derived cardiomyocyte (hPSC‐CM) is powerful tools for disease modelling, drug development, basic research and possible therapies. However, hPSC‐CMs exhibit developmental immaturity that limits these applications. Various researches showed that hPSC‐CM maturation can be enhanced in the ways including: long‐term culturing,^1^ substrate stiffness,^2^ electrical^3^ and mechanical^4^ stimulation, mechanical loading and the interaction with other cell types,^5^ cell patterning and alignment.^6^, ^7^, ^8^ Despite progress in enhancing maturity, these interventions are often time‐consuming, cumbersome or promote only certain aspects of maturation. Cardiomyocytes differentiated from human pluripotent stem cells (hPSC‐CMs) have potential for studying heart disease. Specifically, hPSC‐CMs is a cardiomyocytes (CMs) physiology model in vitro.^9^, ^10^, ^11^ These stem cell–derived cardiomyocytes beat spontaneously, express proteins of sarcomeric and ion channels, and exhibit ventricular‐like action potentials and calcium activity.^12^ hPSC‐CMs have prospect for drug screening and discovery, disease modelling because they adapt to different patients with different genetic backgrounds.^10^ And also, hPSC‐CMs may be a better model than neonatal or mature murine primary CMs in vitro, because they can be maintained in culture longer and the pathophysiology machinery differs between human and murine CMs. However, hPSC‐CMs are immature as they do not display myofibril alignment resembling morphology and sarcomeric protein content and organization like adult cardiomyocytes.^13^, ^14^, ^15^, ^16^ Foetal‐like cardiomyocytes morphology limit their function like the contractility,^17^ intracellular Ca^2+^ handling^15^, ^18^, ^19^ and electrophysiology^20^ of primary adult CMs.^6^, ^7^ Therefore, we need more mature hPSC‐CMs as a research model, which can better simulate the physiological state of human cardiomyocytes.

The foetal cardiomyocytes arrangement is disordered and amorphous, and the adult cardiomyocytes arrangement is more regular in order to adapt to physiological functions such as the strong contraction and electrical conduction of the striated muscle.^21^ CMs alignment is a mature hPSC‐CMs phenotype, which is important for physiological functions of the hPSC‐CMs, such as contractile force and electromechanical coupling.^22^, ^23^ To develop mature hPSC‐CMs via a tissue engineering approach like substrate topography, it is critical to maintain cardiomyocytes and cardiac fibroblasts polarity and alignment.^24^, ^25^, ^26^ Experiments over the last several decades have shown that topographic cues, initially at the microscale and more recently at the nanoscale, can influence the behaviour and differentiation of various cell types.^24^, ^25^, ^26^ Numerous studies of cell growth on flat substrates have demonstrated that cell morphology is extremely responsive to spatial restrictions and topographical order.^26^ An increasing number of studies highlight the sensitivity of morphology and gene expression to micro‐ and nanoscale grooves, pillars or pits. However, engineered multicellular cultures are limited by cell‐to‐cell variations in myocyte type (atrial, ventricular and nodal), cell size, shape and myofibril alignment. And none of these studies has investigated the mechanism by which substrates promote the alignment and maturation of cardiomyocytes.

Mechanical loading is essential to maintain the alignment and function of cardiomyocytes.^27^ And members of the transient receptor potential (TRP) cation channel family are the mechanoreceptors to respond to tension, flow or changes in cell volume.^28^ They play an important role in the remodelling and maintenance of CMs during cardiac development. In previous study, TRPV2 which is the member of the TRP family eliminating in the adult mice, cardiac function declines severely, with disorganization of the intercalated discs.^29^ This article also explains that TRPV2, as an important mechanoreceptor of cardiomyocytes, regulates cardiomyocyte function through potential molecular mechanisms. In our study, we verified the important role of TRP channels in promoting the alignment of cardiomyocytes in the grooves and leading to more mature cardiomyocytes.

In our previous study,^30^ we observed a net‐shaped CM cluster which beat in a certain direction with robust contraction shown developed myofibril structure. To detect the relationship of cell alignment and hPSC‐CMs maturation, we seed hPSC‐CMs on the micron‐scale patterned groove and found that either single CM or cardiomyocytes clusters are oriented along the grooves. This morphological guidance partly mimics the myocardial development, and the TRP channels may involve this process.

## 2 MATERIALS AND METHODS

### 2.1 Fabrication of PDMS

We fabricated 2.5‐dimension microstamp moulds using a 10‐μm layer of negative photoresist (SU‐8 3010, Microchem) on mechanical‐grade, silicon wafers (ø10 cm, University Wafer). Each wafer was mounted on a spin‐coater (Laurel Technologies), and SU‐8‐negative photoresist was added to cover the surface of the wafer. The wafer was spun at 50 *g* for 10 seconds and at 157 *g* for 30 seconds and baked on a hotplate with a temperature cycle of 30°C to 95°C at 4°C/min, 95°C for 6 minutes and 95°C to 30°C at 4°C/min. After incubation in a closed container for 15 minutes at room temperature, the wafer was exposed to 20 mW/cm^2^ light (OAI collimated light source centred at 365 nm) for 10 seconds through a high‐pass ultraviolet filter (PL‐360‐LP, Omega Optical) with the transparency mask described above. Then each wafer was baked on a hotplate with a temperature cycle of 30°C to 95°C at 4°C/min, 95°C for 4 minutes and 95°C to 30°C at 4°C/min. After incubation in a closed container for 15 minutes at room temperature, each wafer was placed in a beaker containing SU‐8 developer (Microchem) for 6 minutes with constant swirling. A stream of new developer was squirted onto the wafers for 15 seconds every minute to enhance removal of undeveloped SU‐8. These SU‐8 microstamp moulds were dried with N_2_ gas and then cast with PDMS. For preparation of PDMS‐184 (Sylgard‐184), prepolymer and curing agent (10:1) were mixed and degassed in a Thinky mixer. PDMS was then poured on the moulds, degassed in a bell jar for 1 hour and cured in a 70°C oven for 24 hours. After curing, the solidified layer of PDMS was carefully peeled from the moulds and stamps areas (1.5 cm × 1.5 cm) were cut from the PDMS layer.

### 2.2 Differentiation, purification and dissociation of hPSC‐CMs

AC‐hPSCs (derived from skin fibroblasts, Cellapy, Beijing, China), NKX2.5eGFP/W‐hESCs (provided by Dr Stanley from Monash University, Australia) and GCaMP‐hESCs, the green fluorescent calcium‐modulated protein 6 fast type (GCaMP) calcium sensor into the AAVS1 locus (provided by Dr Conklin from university of California, San Francisco) were employed in this study. The hPSCs culture and cardiac differentiation methods were described previously. Briefly, hPSCs were maintained in PSCeasy medium (Cellapy). When cells have reached 65%‐85% confluence in 3‐4 days, the cells were treated with 6 µmol/L CHIR99021 (GSK‐3 inhibitor, Selleck Chemical) in basal medium RPMI1640 (Invitrogen) supplemented with B27 for 2 days, then changed to basal medium with the IWP‐2 for another 2 days. The medium is then replaced with the basal medium every other day, and contracting cells will be seen from day 8 to day 9. From day 10 to day 16, RPMI1640 medium without d‐glucose was supplemented with B27 and 5 mmol/L sodium dl‐lactate to metabolically select and purify cardiomyocytes.

The dissociation of CMs was achieved via treatment with CardioEasy CM digestive enzyme (Cellapy) for 30 minutes at 37°C and by pipetting up and down to dislodge the cells and break up the aggregates. The cells were transferred to a 15‐mL conical tube, the remaining volume was filled with the basal medium, and the sample was centrifuged for 5 minutes at 200 *g* at room temperature.

### 2.3 Immunostaining and imaging analyses

For labelling, the cells were fixed in 4% paraformaldehyde, permeabilized in PBS containing 0.2% Triton X‐100 and blocked with 3% BSA. The samples were stained with the following primary antibodies: mouse monoclonal anti‐cardiac troponin T (cTnT) (1:100; Santa Cruz) and rabbit polyclonal anti‐connexin‐43 (1:100, Santa Cruz). After three washes with PBS, the slides were stained for 1 hour with the following secondary antibodies: goat anti‐mouse IgG Alexa Fluor 488, goat anti‐rabbit IgG Alexa Fluor 594, goat anti‐mouse IgG Alexa Fluor 594 and goat anti‐rabbit IgG Alexa Fluor 488 (Invitrogen, these antibodies were used at dilutions of 1:200). All slides were counterstained for 5 minutes with 4,6‐diamidino‐2‐phenylindole (300 nmol/L, Invitrogen).

Bright‐field images were acquired using Toupcam mounted to an Olympus CX41 microscope. Images were quantified using ImageJ software and standard analysis plugins. The cell area, cell perimeter and cell circularity index of each cell were analysed. The circularity index was calculated as 4*πA*/*P*2, where *A* is the area, and *P* is the perimeter.

### 2.4 T‐tubules and mitochondria fluorescent staining

To visualize nascent t‐tubule formation, we used di‐8‐ANEPPS (Life technologies), a lipophilic voltage‐sensitive dye that localizes to t‐tubules in mature hPSC‐CMs. As described previous, we prepared 100 µmol/L di‐8‐ANEPPs in 20% (w/v) Pluronic‐F127 (Sigma) in DMSO from a stock solution of 2 mmol/L di‐8‐ANEPPS in DMSO and added enough cell‐culture medium to incubate cells in 10 µmol/L di‐8‐ANEPPS for 15 minutes at 37°C. The medium was changed, and cells were imaged 30 minutes later. Mitochondria in live cells were labelled with MitoTracker Red (Life Technologies) at a concentration of 50 nmol/L for 10 minutes at 37°C. The cells were washed once with warm PBS, and new warm culture medium was added immediately before imaging. Nuclei were visualized by incubating live cells in medium with 1 µg/mL Hoechst 3342 (Life Technologies) for 5 minutes at 37°C, and fresh medium was added. A confocal microscope (Leica TCS SP5) was used to image t‐tubules labelled with di‐8‐ANEPPS and mitochondrial labelled with mitotracker red.

### 2.5 Calcium transient handling

To image calcium flow, we used GCaMP‐hESC‐derived cardiomyocytes; GCaMP is a calcium‐sensitive modified GFP and thus can be used as a fluorescent reporter under steady‐state level of cytoplasmic Ca^2+^, as previously described,^31^, ^32^ or GCaMP imaging cells were excited with the 488 nm laser line and emission was measured between 505 and 535 nm. Under these conditions, the emitted fluorescence is proportional to the cytosolic‐free calcium concentrations.

To image cell and devices, we used an inverted Leica sp5 microscope equipped with a Hamamatsu C4742‐95 camera. Microscopes had environmental chambers to maintain the temperature at 37°C and CO_2_ level at 5%, fluorescence capabilities and automated stages to save position for time‐lapse studies.

### 2.6 RNA extraction and quantitative real‐time PCR

As described previous, total RNA was extracted with TRIzol (Invitrogen) according to the manufacturer's protocol. For quantitative real‐time PCR, 1 μg of total RNA was reverse transcribed into cDNA using the GoScript reverse transcription system (Promega). The gene expression levels were analysed by quantitative reverse transcriptase PCR (qRT‐PCR) performed with 2× SYBR Master Mix (Takara, Otsu, Shiga, Japan) using an iCycler iQ5 (Bio‐Rad, Hercules, CA, USA). The relative quantification was calculated according to the △C_T_ method. Table S1 shows the primers used.

### 2.7 Video analysis

The cyclic motion of the tissue was analysed on the video acquired from the Olympus CX41. The behaviour of the tissue motion was quantitatively determined from the post‐processing of the acquired video. A cross‐correlation‐based speckle tracking method that is widely used in the field of ultrasound elastography^31^ was applied to the video data to estimate the tissue velocity in this study. The local velocities of the tissue as a function of time were calculated, and the maximum velocities for each cycle and the motion period were determined. The overall motion direction of the tissue at the maximum velocity was obtained by calculating the arctangent of the ratio of horizontal and vertical velocities (downward motion was considered as 0 degree).

### 2.8 Cx43‐localization analysis

Regarding Cx43‐localization within the cell, we determined polar and lateral Cx43 expression as described previously.^33^ Briefly, the longitudinal cell axis was determined and divided into 4 sections of equal length giving 4 areas: the left and right cell pole and the two mid areas. We used ImageJ to measure the length of the plasma membrane (LM) of each section and the length of the immunofluorescence positive membrane (PLM) of the section and calculated the ratio between positively stained membrane length and membrane length PLM section/LM section. The polarity distribution of Cx43 CMs can be quantified by calculating the PML/ML ratio of poles region.

### 2.9 Statistical methods

Data were analysed using the SPSS Statistics 20 (IBM) package and graphed using Prism (GraphPad). The data are presented as (mean ± SEM). Comparisons were conducted using the one‐way ANOVA test, followed by either the All Pairwise Multiple Comparison Procedures (Sidak) method or an unpaired, two‐tailed Student's *t* test.

### 2.10 Western blotting

Cells were lysed using tissue protein extraction reagent (Thermo, USA) containing phosphatase inhibitor cocktail (1:100, Thermo), protease inhibitor cocktail (1:100, Thermo) and 5 mmol/L EDTA (Thermo), and lysates were oscillated and centrifuged (13 000 *g*, 15 minutes, 4°C). The supernatant was collected and stored at −80°C. The protein concentrations were determined using the BCA protein assay kit (Thermo). The protein was mixed with 5× SDS‐PAGE protein loading buffer (Beyotime, China) and denatured by 100°C water bath. Then, the samples denatured were electrophoresed in 10% SDS‐PAGE and transferred to PVDF membranes using transfer device (Bio‐Rad). The membranes were blocked with 5% non‐fat milk prepared in TBST for 1 hour at 37°C and then incubated at 4°C overnight with the primary antibodies: TRPC7 (1:1000, Santa cruz Biotechnology), TRPM4 (1:1000, Santa cruz Biotechnology), TRPC6 (1:1000, Santa cruz Biotechnology), TRPV2 (1:1000, Santa cruz Biotechnology) and the internal normalization mouse anti‐GAPDH (1:1000, Santa cruz Biotechnology). Next, the membranes were washed in TBST, incubated with secondary antibody: Goat anti‐Mouse IgG (H + L) IRDye 800CW or Goat anti‐Mouse IgG (H + L) IRDye 800CW (1:20 000; LI‐COR) for 1 hour at 37°C. The images were observed with a UVA Bio Imaging System and analysed with ImageJ software.

## 3 RESULTS

### 3.1 Micro‐groove remodelling the morphology of immature hPSC‐CMs either single cell or cluster

**FIGURE 1:**
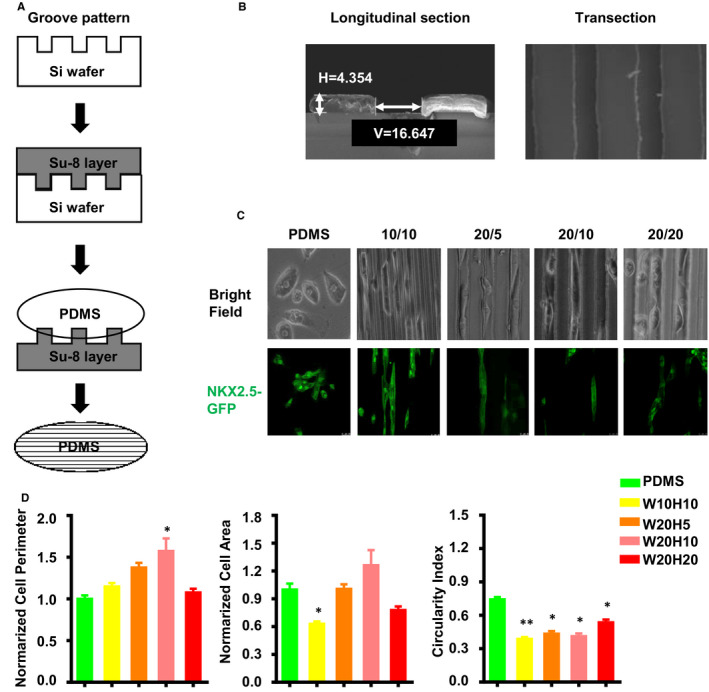
Preparation of micro‐grooves and the effect on the morphology of the single hPSC‐CM. (A) Micro‐grooves' preparation process: Su‐8 photoresist is dropped on the silicon wafer with grooves and processed to obtain the Su‐8 template with grooves; the prepared PDMS is poured onto the Su‐8 template, spin‐coated, solidified and cooled to get PDMS with grooves. (B) Longitudinal section and cross section of a grooved PDMS under an electron microscope. (C) Morphology of the single hPSC‐CMs on the different scale grooves. (D) The statistical graph of the cell perimeter, cell area and circularity index after single cell on the grooves with different parameters (Data are expressed as mean ± standard error, one‐way analysis of variance, *P < .05, **P < .01, ns means no statistical difference, n ≥ 45)

**FIGURE 2:**
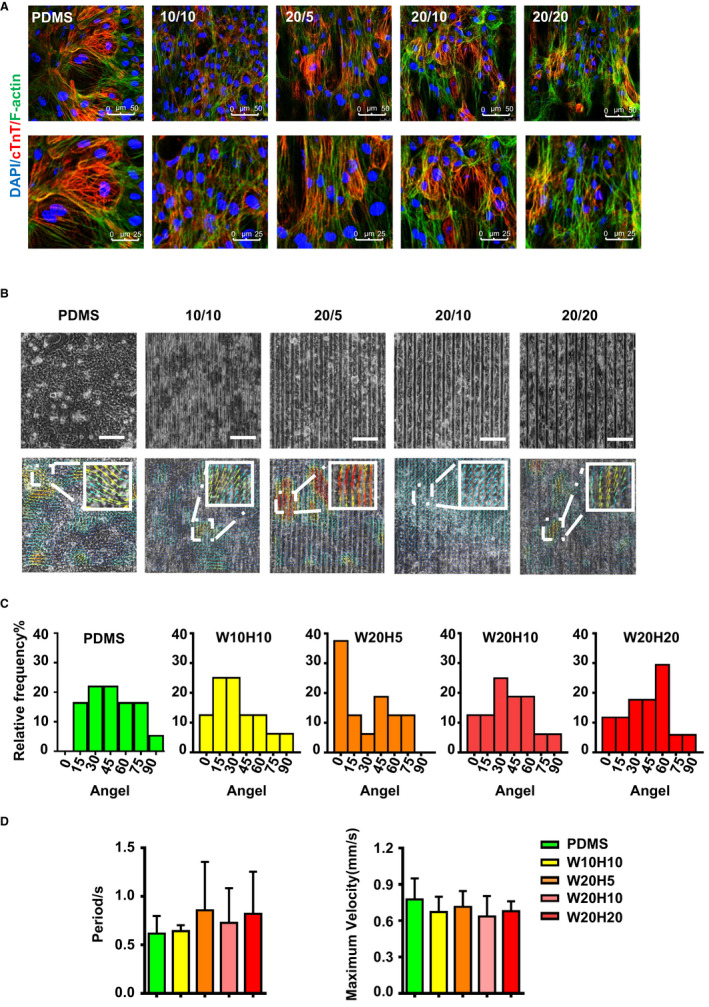
Motion analysis of multi‐hPSC‐CMs on the different scale grooves. (A) Immunofluorescence co‐staining of cardiomyocyte‐specific protein cTnT (red) and phalloidin (green) on the different scale grooves. (B) Motion analysis of hPSC‐CMs on the different scale grooves. The direction of arrows represent the movement direction of hPSC‐CMs, and the colour represents the movement speed. The colour represents the velocity of CMs, red means high velocity and blue means low velocity. The darker the colour, the faster the movement speed. (C) The statistical graph of the movement direction of the (B): angel 0° is along the groove and angel 90° is perpendicular to the groove to evaluate the movement direction of hPSC‐CMs, n = 12. (D) The statistical graph of each contraction time (s) and maximum velocity (mm/s) of the hPSC‐CMs on the different scale grooves, n = 12

To detect the influence of micro‐scaled groove on hPSC‐CM morphology, we seed low cell intensity in different size of grooves, which made in PDMS, and analysed the cellular area, cell perimeter and circularity index. As is shown in Figure 1, after 2 days culturing, compared with PDMS, hPSC‐CMs on became elongated and aligned along the direction of the grooves. W10H10, W20H5 and W20H10 have significant lower circularity index (0.38 ± 0.02, 0.43 ± 0.02, 0.41 ± 0.03 respectively) compared with PDMS (0.74 ± 0.02) (Figure 1D). We also used mitotracker to stain the mitochondria of a single cell on the different grooves, and found that the expression of mitochondria has no obvious difference (Figure S1A). The results indicated that grooves promoted the single hPSC‐CM elongation along the direction of the grooves. Adult cardiomyocytes have cylindrical and parallel structure with regular alignment enables the synchronous contraction of cardiomyocytes. Based on these results, we wonder whether micro‐groove has same effect on high cell intensity. To ensure cell‐cell contact but not cell overlap or single cell growth, we seed cells with the density of 2 * 10^4^ cells on the each 18 mm diameter groove slice. We found that CMs myofilament which cultures on groove was aligned along the groove and sarcomere was perpendicular to the groove. And the W20/H5 seems better than other size grooves (Figure 2A).

### 3.2 Motion analysis of CMs on different grooves

To detect the influence of micro‐scaled groove on hPSC‐CM movement, we used a cross‐correlation‐based speckle tracking method to analyse the movement of the CMs: the maximum velocity, the period of each cycle and the orientation (Figure 2B). To determine whether the direction of movement is significantly changes between different grooves, we divided movement angle into 6 groups from 0 to 90°. The minimum cardiomyocytes orientation value of 0° denoted parallel alignment from the axis of the groove and the maximum of 90° represented perpendicular alignment. As is shown in Figure 2C, the movement direction of hPSC‐CMs on PDMS is irregular, and the movement direction is distributed from 0 to 90°. The minimum value of the motion direction of cardiomyocytes on W10H10 is 3.34°, and the proportion of 0‐15° is 37.5%; the minimum value of cardiomyocytes on W20H5 is 0.30°, and the proportion of 0‐15° is 50%; the minimum value of cardiomyocytes on W20H10 is 1.26°, and the proportion of 0‐15° is 25%. The minimum value of cardiomyocytes movement direction to W20H20 is 3.65°, and the proportion of 0‐15° is 23.4%. The maximum velocity and the period of each cycle were not significantly changed in five groups (PDMS, W10H10, W20H5, W20H10 and W20H20) (Figure 2D). Consequently, cardiomyocytes on PDMS substrate exhibited a wide distribution of cardiomyocytes orientation, but hPSC‐CMs were generally simultaneous contraction in the direction of the grooves. And the cardiomyocytes on W20H5 are order alignment and the movement direction is the most uniformly.

### 3.3 Grooves increase the expression of mature related genes in hPSC‐CMs

**FIGURE 3:**
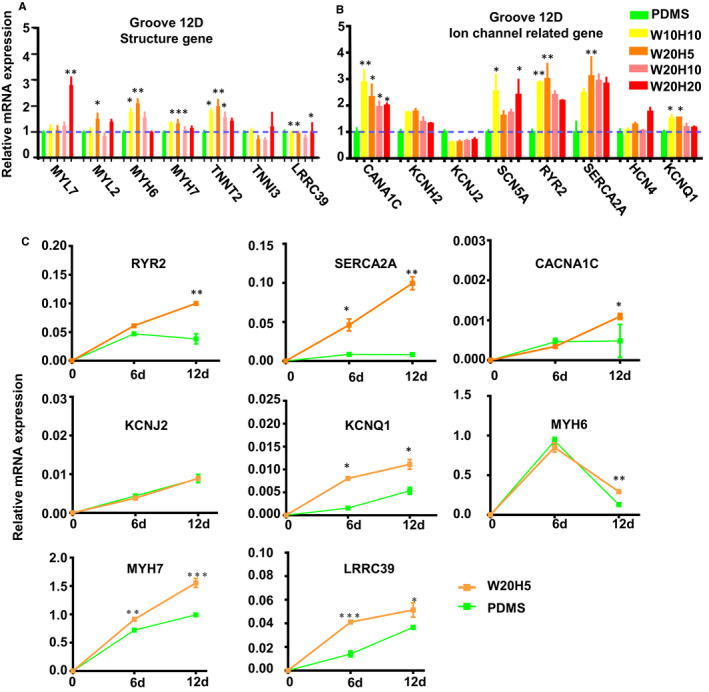
The mRNA expression of maturity‐specific genes of hPSC‐CMs after 12 d culturing on the different scale grooves. (A) The mRNA expression of structural genes of hPSC‐CMs after 12 d culturing on the different scale grooves. (B) The mRNA expression of ion channels genes of hPSC‐CMs after 12 d culturing on the different scale grooves. (C) The mRNA expression of the significantly increasing genes of hPSC‐CMs on the W20H5 grooves from 6 d to 12 d. (The data are expressed as mean ± standard error, one‐way analysis of variance, *P < .05, **P < .01, ***P < .001, n ≥ 3 per group)

Mature cardiomyocytes show changes in a number of genes' expression.^13^ To quantify whether CMs mature, we choose several of genes encoding ion channels and sarcomere protein. We examined the relative expression of those genes using RT‐PCR from NKX2.5‐CMs culturing 6 days on grooves or PDMS. Compared with the PDMS group, the expression of structure genes (MYL2, MYL7, MYH7, TNNT2, TNNI3) and ion channels genes (SCN5A, KCNH2, KNCQ1, KCNJ2, HCN4, LRRC39) in grooves' groups increased, although there is no significant change. The expression of calcium handling genes (CACNA1C, SERCA2A, RYR2) also increased in the grooves' group (Figure S1B). To investigate whether prolonging the time can increase the phenotypes difference between hPSC‐CMs on the W20H5 grooves and PDMS, we examined the expression of mature genes of hPSC‐CMs on W20H5 grooves and PDMS for 12 days. After the hPSC‐CMs were cultured on the grooves for 12 days, compared with the 6 days culturing, the difference of the expression of ion channels (SCN5A, KCNH2, KCNQ1, HCN4, LRRC39) and structure genes (MYL2, MYH6, MYH7, TNNT2, TNNI3) of hPSC‐CMs between the grooves and PDMS was significantly increased (Figure 3A,B). The results show that the expression of calcium handling genes (RYR2, SERCA2A, CANA1C) increased significantly after 12 days culturing on the W20H5 grooves. The expression of mature‐related genes with significantly increasing in W20H5 grooves has been shown in Figure 3C. α‐MHC predominates in the early development of the CMs, and β‐MHC is more robustly expressed in the adult CMs.^34^ Although the expression of α‐MHC (MYH6) of hPSC‐CMs increased on the grooves after 12 days, the increase of the β‐MHC (MYH7) expression was much greater than that of MYH6. And ventricular‐specific expression of myosin light chain‐2V (MYL2; MLC‐2V) also increased on the grooves after 12 days, but not significantly increased. In conclusion, the hPSC‐CMs on the grooves are closer to the mature cardiomyocytes in the expression of sarcomere and ion channels genes. The results indicated that micro‐scaled grooves can promote hPSC‐CMs maturation in transcriptional level.

The results of hPSC‐CMs motion analysis and expression of cardiac maturation genes show that the W20H5 grooves' group performs better than other grooves. Therefore, the W20H5 grooves were used in subsequent studies. Because of the significant changes in the expression of calcium handling genes of W20H5 groups, we further examined the calcium handling function of W20H5 groups.

### 3.4 The W20H5 grooves change hPSC‐CMs function of calcium handling

**FIGURE 4:**
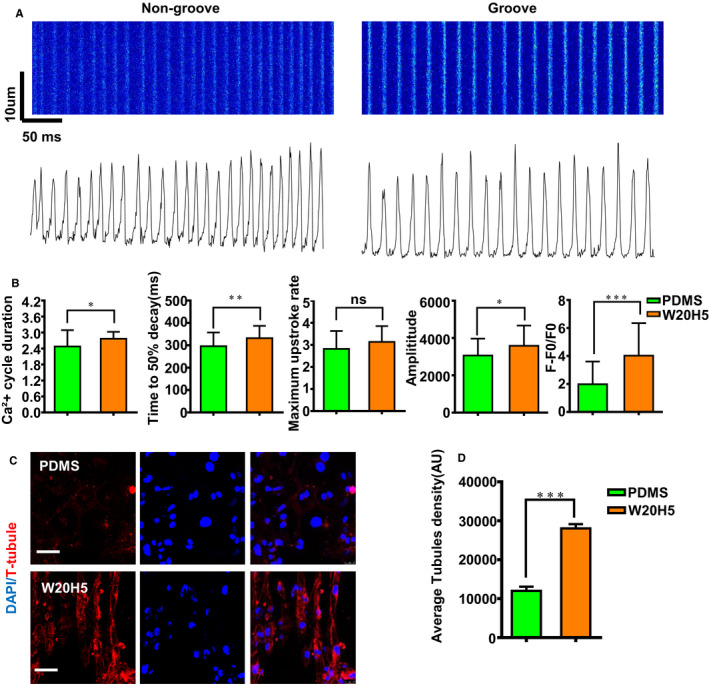
hPSC‐CMs calcium activity on the W20H5 grooves and PDMS. (A) Line scanning (xt‐mode) graph by capturing calcium fluorescence of hPSC‐CMs on both PDMS and W20H5 and the corresponding Ca^2+^ waveform. The upper image is the line scanning graph, and the lower image is the corresponding Ca2 + waveform. (B) The statistical graph from (A) of Ca^2+^ cycle duration, time to 50% decay, maximum upstroke rate, amplitude and F‐F0/F0. (The data are expressed as mean ± standard error, *t* test, *P < .05, **P < .01, ***P < .001, n ≥ 10 in each group.). (C) The staining of the transverse tubules of hPSC‐CMs on the PDMS and W20H5. (D) The fluorescence statistics of the T‐tubules in (C). (The data are shown as the average fluorescence intensity of hPSC‐CMs, expressed as mean ± standard error, analysed by *t* test method, ***P < .001, n ≥ 8 per group)

Mature CMs perform higher calcium storage of SR and faster spontaneous contractions. T‐tubules are critical to effective excitation‐contraction coupling and synchronous triggering of (SR) calcium release in adult CMs, so T‐tubules is an indicator of CMs maturation.^7^, ^35^, ^36^ The grooves promote calcium ion‐channel genes expression, so we speculated that the W20H5 grooves can improve hPSC‐CMs calcium handling. AAVS1‐GCaMP (hESCs‐AAVS1‐GCaMP) was performed to detect the effect of groove to CMs calcium handling.^37^ To further profile the calcium imaging results, calcium fluorescence data were analysed using Metalab software, including basic values of calcium fluorescence, peaks of calcium releasing, maximum speed of calcium transients, and 50% of the time for calcium to decay (Figure 4A). Calcium imaging revealed faster spontaneous contractions in the W20H5 group, which reflected in the increase of Ca^2+^ cycle duration, time to 50% decay. Compared with the PDMS group, the W20H5 group showed higher calcium release amplitude and F‐F0/F0 (Figure 4B), suggesting that higher calcium storage of SR in the W20H5 group than the PDMS group. To investigate the calcium handling function, we performed immunofluorescence staining on the T‐tubules. The results show that hPSC‐CMs on the PDMS hPSC‐CMs have no obvious T‐tubule structure on PDMS, but hPSC‐CMs on the W20H5 had T‐tubule‐like structures distributed along the cell membrane (Figure 4C,D). As a consequence, the CMs on the W20H5 show more mature calcium handling function.

### 3.5 Polarity distribution of Cx43 on the W20H5 grooves

**FIGURE 5:**
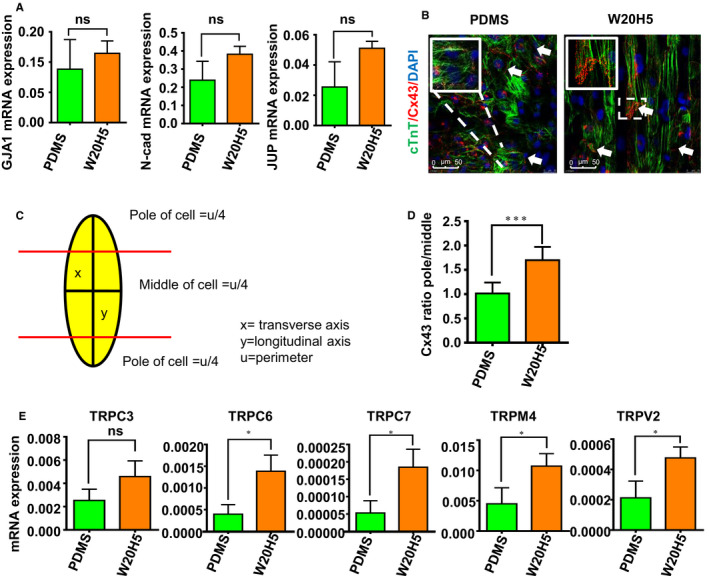
W20H5 grooves promote cell‐polar distribution of connexin43 (CX43). (A) The mRNA expression of gap junction genes (GJA1, N‐cadherin and JUP) of hPSC‐CMs on the PDMS and W20H5 grooves (The data are expressed as mean ± standard error, one‐way analysis of variance, *P < .05, **P < .01, ***P < .001, n ≥ 3 per group) (B) Immunofluorescence staining of hPSC‐CMs proteins of cTnT and CX43 on the W20H5 grooves and PDMS (green is cTnT, red is CX43, and blue is DAPI). (C) The schematic diagram of the quantification of CX43 distribution on the cell membrane. Dividing the longitudinal cells into 4 areas, as shown in the figure, the upper and lower cell poles and the middle area, we use image J software to calculate the membrane length (LM) of each area and the CX43‐positive membrane length (PLM) of the area. By calculating the ratio of PLM/LM in each area, the polar distribution of CX43 cells can be quantified. (D) The statistical results of the polarity distribution of CX43 from (B) (the data are expressed as the mean ± standard error, analysed by the *t* test method, ***P < .05, n = 16.). (E) The mRNA expression of TRP channels (TRPC3, TRPC6, TRPC7, TRPM4, TRPV2) of hPSC‐CMs on the PDMS and W20H5 grooves. (The data are expressed as mean ± standard error, one‐way analysis of variance, *P < .05, **P < .01, ***P < .001, n ≥ 3 per group)

The intercalated disc (ID) at the longitudinal cell edges of cardiomyocytes provides as a macromolecular infrastructure that integrates mechanical and electrical coupling within the heart. Compared to the foetal cardiomyocytes, connexin43 (Cx43) tends to polar distribution in adult.^7^, ^33^, ^38^ To determine the effect of the substratum micro‐scaled groove on the expression of connection protein typically found in the heart, we examined the genes expression of the ID from the PDMS and W20H5 group. The expression of Cx43, JUP and N‐cadherin increased but has no statistical significance (Figure 5A). And also we did a protein level verification; the results showed that the expression of Cx43 has no statistical significance (Figure S2A). However, it is interesting that we found the distribution of Cx43 protein has a certain pattern. As is shown in Figure 5B, hPSC‐CMs perform polarity distribution of Cx43 on W20H5 grooves (Figure 5B). The quantification of the Cx43 distribution is based on the quantification methods reported in the literature,^33^ as shown in Figure 5C. The results show that Cx43 was preferentially accentuated at the cell pole, compared to the PDMS group performing a more random distribution (Figure 5B,D). We supposed the grooves play a role as mechanical stimulation and stretching stimulation in hPSC‐CMs maturing process.

### 3.6 TRP channels participate in the W20H5 grooves‐promoting hPSC‐CMs maturity

**FIGURE 6:**
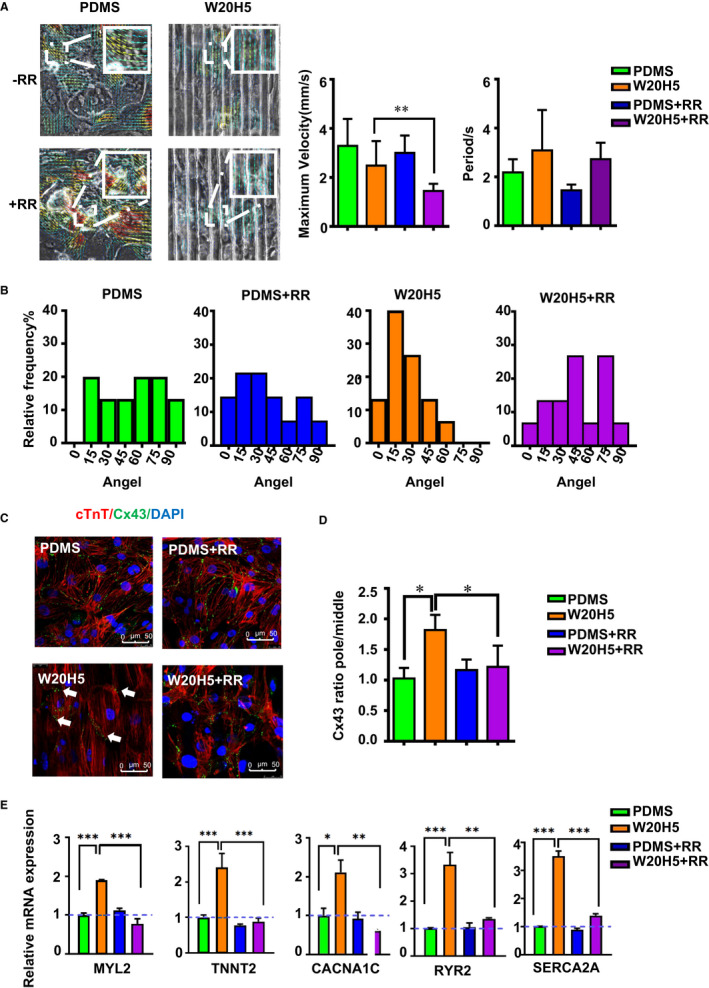
TRP channels participate in the W20H5 grooves‐promoting hPSC‐CMs maturity through cell‐polar distribution of connexin43 (CX43). (A) Motion analysis of hPSC‐CMs on the W20H5 grooves and PDMS with or without RR. The direction of arrows represented the movement direction of hPSC‐CMs, and the colour represents the movement speed. The colour represents the velocity of CMs, red means high velocity, and blue means low velocity. The darker the colour, the faster the movement speed. (B) The statistical graph of the movement direction of the (A), n = 12. (C) Immunofluorescence staining of hPSC‐CMs proteins of cTnT and CX43 on the W20H5 grooves and PDMS with or without RR (green is cTnT, red is CX43, and blue is DAPI). (D) The statistical results of the polarity distribution of CX43 from (C) (the data are expressed as the mean ± standard error, analysed by the *t* test method, ***P < .05, n = 16.) (E) The mRNA expression of maturity‐specific genes of hPSC‐CMs on the W20H5 grooves and PDMS with or without RR. (The data are expressed as mean ± standard error, one‐way analysis of variance, *P < .05, **P < .01, ***P < .001, n ≥ 3 per group)

TRP channels as non‐selective cationic channels are important in major physiological processes in the heart.^28^, ^39^ In the adult heart, TRP channels as a mechanoreceptor of the heart regulate the physiological function of the heart by sensing haemodynamic stimulation.^28^, ^39^ We supposed that the TRP channels affect the alignment of cardiomyocytes by sensing mechanical stimulation, thereby affecting the maturation of cardiomyocytes. To study the mechanism of grooves‐promoting cardiomyocytes alignment orderly, we examined the expression of TRP relative genes on the PDMS group and the W20H5 group. The result showed that gene expression of TRPC6, TRPC7, TRPM4 and TRPV2 is significantly increased and no significant change in gene expression of TRPC3 (Figure 5E). We did a protein level verification to further determine the specific TRP channels worked, and the result showed that the expression of TRPV2 increased after hPSC‐CMs seeding on the groove W20H5 12 days (Figure S2B). To further profile the TRP function in grooves, we used ruthenium red (RR)—a classic non‐specific inhibitor of the TRP channel. We treated the hPSC‐CMs with RR of 10 μmol/L and analysed the motion of the four groups (PDMS with or without RR, W20H5 with or without RR) CMs with a cross‐correlation‐based speckle tracking method. To determine the effect of RR on hPSC‐CMs, we did a calcium fluorescence experiment which showed after adding RR calcium fluorescence, intensity was significantly reduced (Figure S2C‐E). We also added APB, the agonist of TRP channels, and the calcium fluorescence intensity increased significantly (Figure S2C‐E). The result of the motion analysis showed that, compared with the W20H5 group, the hPSC‐CMs with W20H5 + RR treatment group performed a significantly lower maximum exercise speed. But the W20H5 + RR group behaved no significant difference with the PDMS group or the PDMS + RR group. RR did not significantly change in the heart rate of both the W20H5 and PDMS group of hPSC‐CMs (Figure 6A,B). And we analysed the angle of motion direction; similar to previous results, compared to the W20H5 group, the motion direction of hPSC‐CMs of the PDMS group is more irregular. The results show that the minimum value of the motion direction of W20H5 is 6.82°, and the proportion of 0‐15° is 50%; the motion direction of PDMS equally distributed from 0 to 90°, and the proportion of 0‐15° is 20%. The PDMS + RR group has no regular motion direction of the hPSC‐CMs, the minimum value of the movement direction is 1.81°, and the proportion of 0‐15° is 20%. Compared with the W20H5 group and the PDMS + RR group, the proportion of vertical movement direction of the W20H5 + RR group is less than W20H5 group, but more than PDMS + RR group. The minimum value of W20H5 with RR group is 1.09°, and the proportion of 0‐15° is 38% (Figure 6A,B). The TRP channels are the mechanism of hPSC‐CMs aligning in response to the grooves' topography. Moreover, the lack of TRP channels will cause the CMs failing to contract in the same direction, which may affect the transmission of force.

### 3.7 TRP inhibitor inhibiting polarity distribution of Cx43

In the previous results, we found that the W20H5 grooves can promote the polarity distribution of Cx43. We speculated whether TRP channels can promote the alignment of hPSC‐CMs by affecting the polarity distribution of Cx43. To further study the mechanism of TRP channels causing hPSC‐CMs alignment, we used immunofluorescence to stain hPSC‐CMs of cTnT and Cx43. The Cx43 of the W20H5 group expressed mainly concentrated at both poles of the CMs, and muscle fibres of CMs of W20H5 group are arranged along the grooves' direction, although Cx43 expressed no significant difference between the PDMS group and the PDMS + RR. And Cx43 in the W20H5 + RR group was also distributed at the CMs' membrane surface, but did not express at both poles of the CMs. And compared to the W20H5 group, muscle fibres of hPSC‐CMs of the W20H5 + RR group did not align along the grooves' direction. We used imageJ to quantify the Cx43 polarity distribution. The results show that comparing to W20H5, the Cx43 polarity distribution of the W20H5 + RR group is significantly reduced and statistically decreased, although it is not significantly different from the PDMS group and PDMS + RR group (Figure 6D). The results suggest that the muscle fibres aligning in the direction of grooves and the polarity distribution of Cx43 were related to TRP channels. The previous results show that inhibiting TRP channels on the grooves will cause CMs' muscle fibres aligning and moving irregularly, and Cx43 is not polar distribution, which are the key factors affecting hPSC‐CMs maturity. To further verify the hPSC‐CMs maturity after inhibiting TRP channels, we used RT‐PCR to examine the expression of mature‐related genes in the following four groups. As is shown in the Figure 6E, compared to the W20H5 group, the hPSC‐CMs mature marker genes (MYL2, TNNT2, CANA1C, SERCA2A, RYR2) were significantly higher than those in the PDMS group and the PDMS + RR group. The expression level of the hPSC‐CMs mature marker genes in the W20H5 + RR group was significantly lower than that in the W20H5 group, which was statistically significant different. Although there was no significant difference of the expression of mature‐related genes between the PDMS group and the PDMS + RR groups, the results show that TRP channels were involved in the process of groove‐promoting hPSC‐CMs maturity.

## 4 DISCUSSION

In this study, we mainly made grooves to promote the maturation of hPSC‐CMs by aligning the muscle fibres of hPSC‐CMs in a regular arrangement and contracting in the same direction. And the hPSC‐CMs maturity phenotype in the groove of W20H5 is the most obvious, showing that the direction of movement is closest to the direction of hPSC‐CMs alignment, the muscle fibres arranging most regularly, the peak of calcium releasing and the expression of T‐tubules increasing, and Cx43 showing a polar distribution. In addition, we explored the mechanism of groove‐promoting hPSC‐CMs maturation. Cardiomyocytes senses force stimulation through the TRP channel.^28^, ^39^ Specifically, we show that hPSC‐CMs through TRP channels promote the polarity distribution of Cx43 and the orderly arrangement of muscle fibres. We used the Cx43 distribution as a key indicator of hPSC‐CMs maturity and the crucial phenotype of exploring groove‐promoting maturity mechanism.^7^, ^40^ Our research reveals that micro‐scaled grooves can promote hPSC‐CMs maturation, and we have studied the mechanisms of grooves‐promoting hPSC‐CMs maturation.

By seeding few cardiomyocytes on micro‐scaled grooves, it was observed that the grooves would change the morphology of an individual CM and cause the CMs stretching in the groove direction (Figure 1B,C). With the change of CMs physiological morphology, the cells' function also tends to mature phenotypes. Compared to single cells, cell‐to‐cell electrical and mechanical conduction are very important physiological functions. We observed that the arrangement and direction of movement of CMs on the grooves were regular (Figure 2B‐D). In particular, the percentage of movement direction of CMs on the W20H5 groove between 0 and 15 degrees is 50%, which is more than that on other grooves (Figure 2C). However, the maximum speed of CMs on the W20H5 motion was not significantly different from PDMS (Figure 2D). This may be due to the limitations of the micron‐grooves itself. On the W20H5 grooves, hPSC‐CMs' morphological change is reflected on the alignment of muscle fibres and cell lengthening closer to the adult cardiomyocytes morphology. The cellular function of primary CMs depends on these intramuscular and extramuscular cytoskeletal structures.^41^ Stress plays a very important role in myocardial alignment during the process of myocardial maturation. In our results, the W20H5 grooves play a role as applying pressure in vitro in aligning the CMs.

In order to further verify whether the grooves will promote the maturation of CMs, we choose some gene expression of maturity.^42^ The cardiomyocytes were seeded on the grooves for 6 days and 12 days to determine the relative gene expression of maturity (Figure 3A‐D). The expression of maturity genes of CMs on the grooves for a week increased, but there was no significant difference until 2 weeks of culturing, indicating that it takes at least 2 weeks for structural changing to affect the function. And the group of W20H5 express the mature genes^42^ like MYH7, TNNT2, KCNH2, RYR2, SERCA2A, KCNQ1 were higher than other groove groups (Figure 3C,D). So we chose the W20H5 groove for subsequent experiments. And also W20H5 grooves promote ion channels relative gene expression especially expression of calcium channels, like CACNA1C, RYR2 and SERCA2A (Figure 3E). Because the muscle fibres aligned on the W20H5 grooves, the function of hPSC‐CMs becomes more mature. The most significant change is the gene expression related to calcium treatment. We further verified the calcium treatment function of myocardial cells whether consistent with gene expression results.

One of the most widely characterized functional phenotypes in hPSC‐CMs is calcium handling. Ca2 + influx from extracellular via L‐type Ca2 + channels as an initial trigger initiates Ca2 + release from the sarcoplasmic reticulum by activating Ryanodine receptor 2 (RyR2) via a process called calcium‐induced calcium release (CICR).^43^, ^44^, ^45^ Increased function of these channels leads to increased calcium influx, increasing contractility. In order to further verify whether the cardiomyocytes on the W20H5 groove are functionally more mature than on the PDMS, we performed a verification of calcium fluorescence, in which shows that the hPSC‐CMs on the W20H5 grooves have a higher spontaneous beating frequency and greater calcium transient amplitudes. The spontaneous calcium cycling is faster in the W20H5 group, which could correspond with the polar distribution of Cx43. And hPSC‐CMs with these calcium imaging phenotypes are more mature. The formation of T‐tubules is one of the important indicators of hPSC‐CMs maturity.^35^, ^36^ The hPSC‐CMs culturing on the PDMS absent of the structure of the T‐tubules, corresponding with the previous studies, hPSC‐CMs have few to no T‐tubules in 2D culture.^32^, ^42^, ^43^ And hPSC‐CMs growing on the grooves could obviously observe the immunofluorescence staining of the T‐tubules (Figure 4C,D). This further corresponds to the calcium fluorescence of the front results and the hPSC‐CMs on W20H5 grooves are more mature of the calcium handling function.

In terms of hPSC‐CMs maturity, Cx43 is also an important indicator, not only in the expression of Cx43 but also in the distribution of Cx43.^13^ Cx43 tends to be distributed at both ends of the mature CMs (Figure 5B,D). The polarity distribution of Cx43 on the W20H5 grooves confirms this conclusion. We made a reasonable suppose that the polarity distribution of Cx43 is the mechanism of the W20H5 grooves promoting hPSC‐CMs alignment and maturation. We chose the obvious difference—the distribution of Cx43 on the W20H5 to explain the phenomenon of neat arrangement of muscle fibres. However, whether only Cx43 plays a role in this process requires further study.

In previous studies of the grooves, the maturation‐promoting phenotype of grooves has been fully studied, but little research has been done on the mechanism. Cx43 distributes to the poles of CMs through the external force providing from the W20H5 groove. So, we supposed that mechanoreceptor of CMs plays an important role in the process of hPSC‐CMs aligning. TRP channels are an important family of cation channels located on the cell membrane.^28^ They play an important role in the remodelling and maintenance of CMs during cardiac development. In this study, we used RR which are typical TRP channel inhibitors and non‐selective inhibitors of TRPV2 in rodent hearts.^29^ After RR treated, RR inhibited the distribution of Cx43 in the cell poles. This is consistent with the phenotype of TRPV2‐specific heart tissue‐specific knockout mice. The intercalated discs' structure was deleted after TRPV2 knockout, and the distribution of Cx43 was abnormal.^29^ TRP channels participate in the process of maturation of the grooves by affecting the distribution of Cx43.

In conclusion, there still are some limitations. TRP channels include six types of channels: TRPC, TRPV, TRPM, TRPA, TRPP and TRPML. It is still unclear whether a certain type of channel or the combination of several major channels is in the process of promoting hPSC‐CMs maturation. However, we tested the protein level of TRP channels between the group of PDMS and W20H5 in order to select specific mechanoreceptor channels to further verify their functions. And the result showed that the expression of TRPV2 increased after hPSC‐CMs seeding on the groove W20H5 12 days (Figure S2B). To this end, we further apply specific inhibitors known for TRP channels or combine RNAseq with literature research to find the most likely channels and establish knockout cell lines. Results in this paper revealed that TRP channels may participate in the process of promoting maturation of the groove by affecting the cell distribution of Cx43, but how the TRP channels affect the distribution of Cx43 and whether it directly or not interacts with Cx43 protein is still unclear. Therefore, we next use co‐immunoprecipitation method for further study.

## CONFLICT OF INTEREST

The authors declare that there is no conflict of interest in relation to the experiments or this paper.

## AUTHOR CONTRIBUTIONS

**Taoyan Liu:** Data curation (lead); resources (lead). **Siyao Zhang:** Data curation (equal); writing‐original draft (lead). **Chenwu Huang:** Data curation (equal); software (equal). **Shuhong Ma:** Formal analysis (equal); validation (equal); writing‐original draft (equal). **Rui Bai:** Formal analysis (equal); validation (equal). **Yanan Li:** Data curation (equal); validation (equal); visualization (equal). **Yun Chang:** Investigation (equal); validation (equal). **Chenwen Hang:** Data curation (equal); validation (equal); visualization (equal). **Amina Saleem:** Data curation (equal); writing‐original draft (equal). **Tao Dong:** Data curation (equal); validation (equal). **Tianwei Guo:** Formal analysis (equal); validation (equal). **Youxu Jiang:** Data curation (equal); validation (equal). **Wenjing Lu:** Investigation (equal); validation (equal). **Lina Zhang:** Formal analysis (equal); software (equal). **Luo Jianwen:** Data curation (equal); resources (equal). **Hongfeng Jiang:** Data curation (lead); resources (lead). **Feng Lan:** Funding acquisition (supporting); project administration (supporting); writing‐review & editing (lead).

## Supporting information

Figure S1

Figure S2

Table S1

Supplementary Material
