# Supplementary figures and images for: Microscale grooves regulate maturation development of hPSC‐CMs by the transient receptor potential channels (TRP channels)
Source: J Cell Mol Med. 2021 Mar 10;25(7):3469–83. doi: 10.1111/jcmm.16429 (PMC8034460; doi:10.1111/jcmm.16429)

A

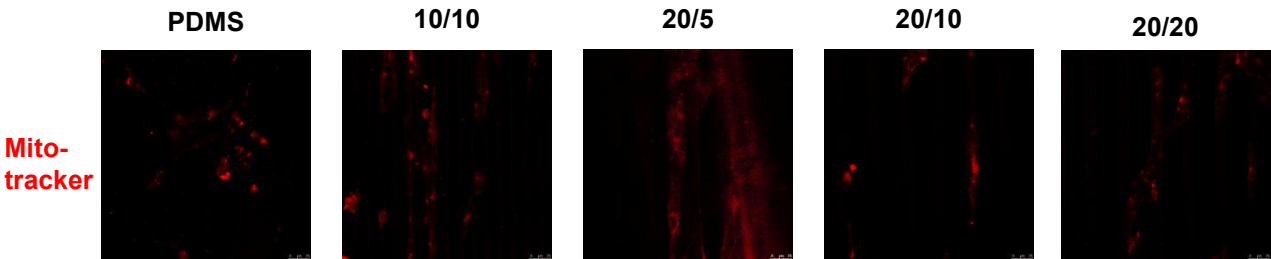

B

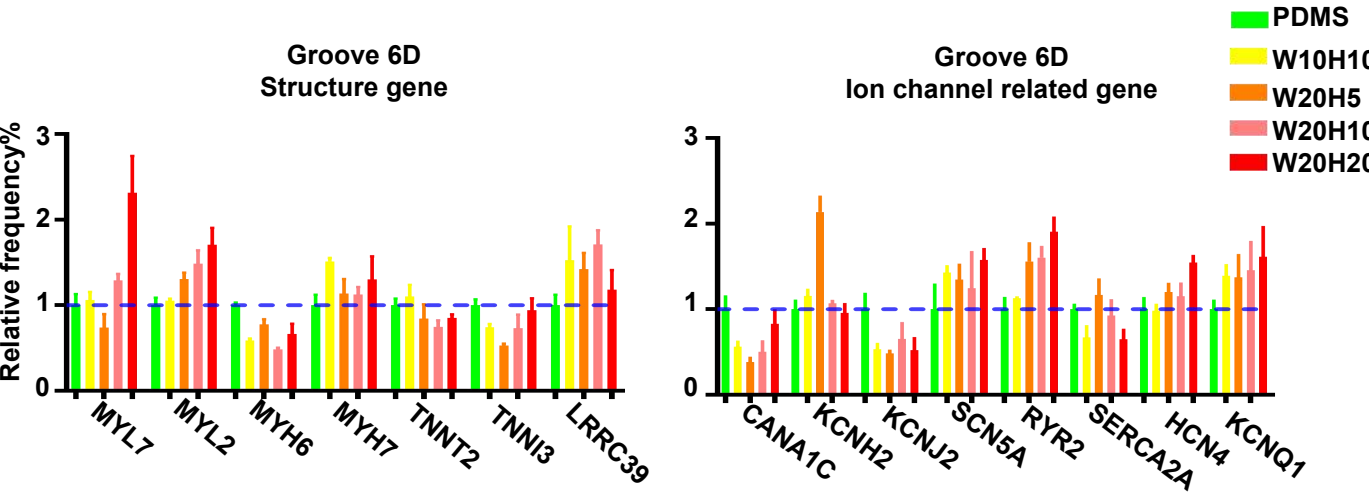

Supplement: Supplementary file 1 — Figure S1 [file JCMM-25-3469-s001.pdf]

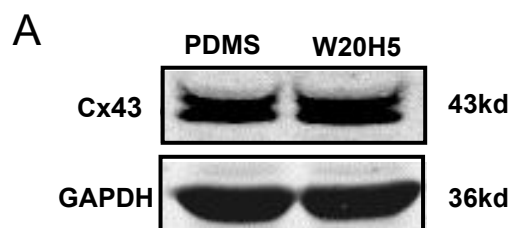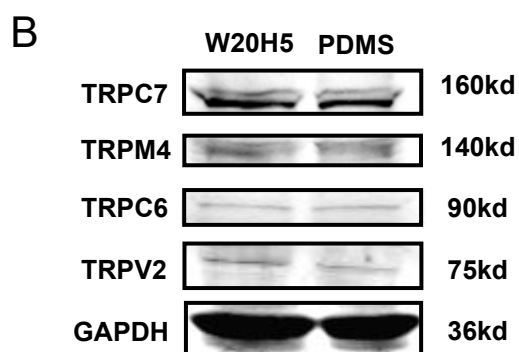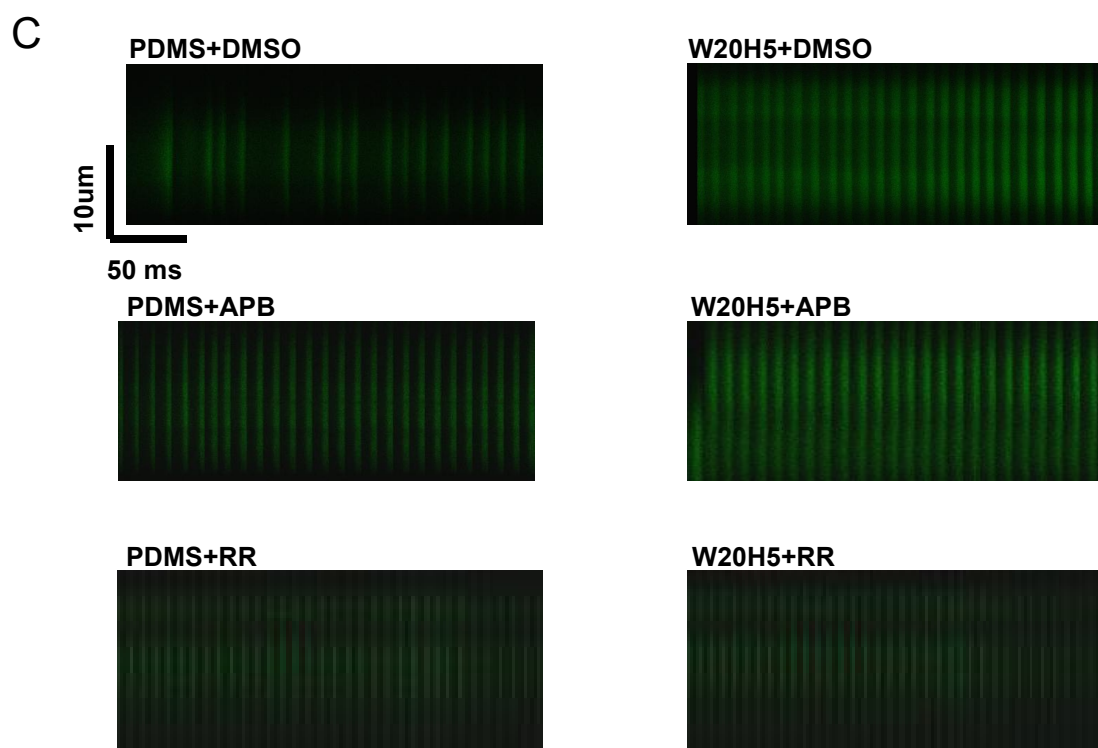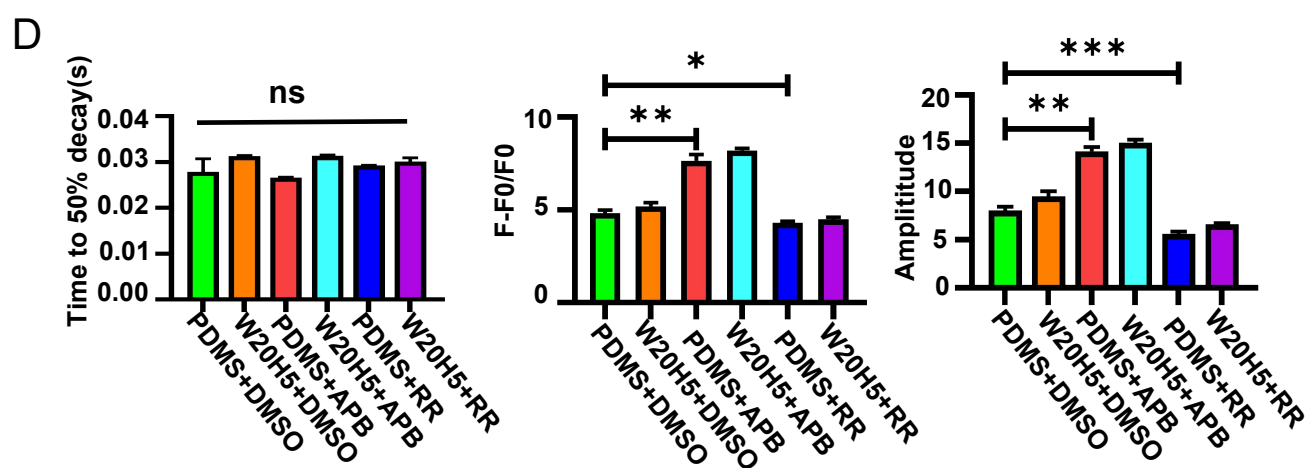

Supplement: Supplementary file 2 — Figure S2 [file JCMM-25-3469-s003.pdf]
