# Supplementary material for: Microscale grooves regulate maturation development of hPSC‐CMs by the transient receptor potential channels (TRP channels)
Source: J Cell Mol Med. 2021 Mar 10;25(7):3469–83. doi: 10.1111/jcmm.16429 (PMC8034460; doi:10.1111/jcmm.16429)
Supplement: Supplementary file 3 — Table S1 [file JCMM-25-3469-s002.pdf]

TABLE S1：Primer sequence used for qPCR

Gene	Orientation	Primer sequence(5' to 3')
GAPDH	Forward	GTGGACCTGACCTGCCGTCT
GAPDH	Reverse	GGAGGAGTGGGTGTCGCTGT
TNNT2	Forward	TTCACCAAAGATCTGCTCCTCGCT
TNNT2	Reverse	TTATTACTGGTGTGGAGTGGGTGTGG
MYH6	Forward	TCTCCGACAACGCCTATCAGTAC
MYH6	Reverse	GTCACCTATGGCTGCAATGCT
MYH7	Forward	GGCAAGACAGTGACCGTGAAG
MYH7	Reverse	CGTAGCGATCCTTGAGGTTGTA
MYH7	Forward	GGGCAACAGGAAAGTTGGC
MYH7	Reverse	ACGGTGGTCTCTCCTTGGG
MYL2	Forward	TACGTTCGGGAAATGCTGAC
MYL2	Reverse	TTCTCCGTGGGTGATGATG
MYL7	Forward	CCGTCTTCCTCACGCTCTT
MYL7	Reverse	TGAACTCATCCTTGTTCACCAC
LRRC39	Forward	CTGGGTACTCTTGTTCTCAG
LRRC39	Reverse	TCCCGTTCCTCTTCTTCATC
KCNH2	Forward	TTCGACCTGCTCATCTTCGG
KCNH2	Reverse	CGATGCGTGAGTCCATGTGT
SCN5A	Forward	TCTCTATGGCAATCCACCCCA
SCN5A	Reverse	GAGGACATACAAGGCGTTGGT
JUP	Forward	ACACCTACGACTCGGGTATC
JUP	Reverse	CTGGCTGTTGTGGACATCTG
GJA1	Forward	CAATCACTTGGCGTGACTTC
GJA1	Reverse	AAAGGCAGACTGCTCATCTC
CACNA1C	Forward	CAGAGGCTACGATTTGAGGA
CACNA1C	Reverse	GCTTCACAAAGAGGTCGTGT
RYR2	Forward	AGAACTTACACACGCGACCTG
RYR2	Reverse	CATCTCTAACCGGACCATACTGC
E-cadherin	Forward	AATTCCTGCCATTCTGGGGA
E-cadherin	Reverse	TCTTCTCCGCCTCCTTCTTC
N-cadherin	Forward	TGAGCCTGAAGCCAACCTTA

N-cadherin	Reverse	AGGTCCCCTGGAGTTTTCTG
HCN4	Forward	GAACAGGAGAGGGTCAAGTCG
HCN4	Reverse	CATTGAAGACAATCCAGGGTGT
KCNQ1	Forward	GCGTCTCCATCTACAGCACG
KCNQ1	Reverse	GAAGTGGTAAACGAAGCATTTCC
ATP2A2	Forward	TGGGTGTATGGCAGGAAAGAA
ATP2A2	Reverse	ACTGGTCAACTCTTAGTGTGGTA
TNNI3	Forward	TTTGACCTTCGAGGCAAGTTT
TNNI3	Reverse	CCCGGTTTTCCTTCTCGGTG
KCNJ2	Forward	CTGGCTTTCGTCCTGTCATGG
KCNJ2	Reverse	GCCCACGATTGACTGGAACA
