# Supplementary material for: Microscale grooves regulate maturation development of hPSC‐CMs by the transient receptor potential channels (TRP channels)
Source: J Cell Mol Med. 2021 Mar 10;25(7):3469–83. doi: 10.1111/jcmm.16429 (PMC8034460; doi:10.1111/jcmm.16429)
Supplement: Supplementary file 4 — Supplementary Material [file JCMM-25-3469-s004.docx]

Figure S1. (A) Single hPSC-CM immunofluorescence staining with mitochondria on the different scale grooves. (B) The mRNA expression of maturity specific genes of hPSC-CMs after 6 days culturing on the different scale grooves.

Figure S2. (A) Cx43 protein expression of hPSC-CMs after 12 days culturing on the PDMS and W20H5. (B) TRPC7,TRPM4,TRPC6,TRPV2 protein expression of hPSC-CMs after 12 days culturing on the PDMS and W20H5. (C) The calcium fluorescence of hPSC-CMs on both PDMS and W20H5 and the corresponding Ca2+ waveform. (D) The statistical graph from supplementary 2 of time to 50% decay, amplitude and F-F0/F0. (The data are expressed as mean ± standard error, t-test test, *P<0.05, **P<0.01, ** *P<0.001, n≥10 in each group.)

TABLE S1.The primer sequence used for qPCR
